# Infection Elicited Autoimmunity and Myalgic Encephalomyelitis/Chronic Fatigue Syndrome: An Explanatory Model

**DOI:** 10.3389/fimmu.2018.00229

**Published:** 2018-02-15

**Authors:** Jonas Blomberg, Carl-Gerhard Gottfries, Amal Elfaitouri, Muhammad Rizwan, Anders Rosén

**Affiliations:** ^1^Department of Medical Sciences, Uppsala University, Clinical Microbiology, Academic Hospital, Uppsala, Sweden; ^2^Gottfries Clinic AB, Mölndal, Sweden; ^3^Department of Infectious Disease and Tropical Medicine, Faculty of Public Health, Benghazi University, Benghazi, Libya; ^4^Department of Clinical and Experimental Medicine, Division of Cell Biology, Linköping University, Linköping, Sweden

**Keywords:** chronic fatigue syndrome, myalgic encephalomyelitis, irritable bowel syndrome, postexertional malaise, autoimmunity

## Abstract

Myalgic encephalomyelitis (ME) often also called chronic fatigue syndrome (ME/CFS) is a common, debilitating, disease of unknown origin. Although a subject of controversy and a considerable scientific literature, we think that a solid understanding of ME/CFS pathogenesis is emerging. In this study, we compiled recent findings and placed them in the context of the clinical picture and natural history of the disease. A pattern emerged, giving rise to an explanatory model. ME/CFS often starts after or during an infection. A logical explanation is that the infection initiates an autoreactive process, which affects several functions, including brain and energy metabolism. According to our model for ME/CFS pathogenesis, patients with a genetic predisposition and dysbiosis experience a gradual development of B cell clones prone to autoreactivity. Under normal circumstances these B cell offsprings would have led to tolerance. Subsequent exogenous microbial exposition (triggering) can lead to comorbidities such as fibromyalgia, thyroid disorder, and orthostatic hypotension. A decisive infectious trigger may then lead to immunization against autoantigens involved in aerobic energy production and/or hormone receptors and ion channel proteins, producing postexertional malaise and ME/CFS, affecting both muscle and brain. In principle, cloning and sequencing of immunoglobulin variable domains could reveal the evolution of pathogenic clones. Although evidence consistent with the model accumulated in recent years, there are several missing links in it. Hopefully, the hypothesis generates testable propositions that can augment the understanding of the pathogenesis of ME/CFS.

## Introduction

ME/CFS is a common disease of unknown etiology characterized by postexertional malaise (PEM; a type of fatigability), cognitive disturbance, unrefreshing sleep, autonomic nerve dysfunction, and a few characteristic comorbidities, see, e.g., Ref. (1–3). It often starts with an infection and has a strong tendency to remain a chronic condition.

ME/CFS diagnostic criteria have gradually become more stringent, see, e.g., Ref. (2–7). These are based on somatic, often self-reported symptoms (8, 9). Although often used interchangeably, studies using the “CDC,” (also called the “Fukuda”) criteria (5) mainly use the term “CFS,” while those using the “Canada” (3) or International consensus (2) criteria use the term “ME.” This creates an ambiguity, which may explain some contradicting results. There are so far no specific laboratory tests (10) for ME/CFS diagnosis. Recently, a committee recommended a new name for ME/CFS, systemic exhaustion intolerance disease (SEID) (11, 12) with diagnostic criteria that emphasize PEM as the central ME/CFS symptom (13). The disease entity ME/CFS is not uncontroversial. Like many times before in medical history, psychiatric and somatic explanations compete with each other. A recent critical review, which emphasized psychiatric aspects, stated that “there is no convincing pathogenesis model for CFS” (14). However, in this review, we forward that evidence for a somatic origin of the disease is accumulating.

From a research perspective it is important that patients are diagnosed using strict criteria. A thorough clinical examination is necessary. It does not matter how sophisticated the analyses are in a study if patient selection is ambiguous. In the case of “fatigue” it is important to distinguish ME/CFS fatigue from other types of fatigue, such as burnout syndrome and depression, see, e.g., Ref. (15). In ME/CFS, repetition of a physical or mental exertion can reveal objective evidence of fatigability. This exertion-elicited fatigue, PEM, is required for the diagnosis of ME/CFS using the Canadian criteria (3), the International consensus (2), and the SEID (12), but not using CDC (5) criteria. Although the term “ME/CFS,” encompassing both “ME” and “CFS,” has a built-in ambiguity it covers much of the current studies and is operationally judged as the best available concept. Fatigue similar to PEM also occurs in Sjögren’s syndrome (SS), primary biliary cholangitis (also named primary biliary cirrhosis) (PBC), and systemic lupus erythematosus (SLE). The relation of ME/CFS to the similar condition Gulf War Illness (GWI) is uncertain, see, e.g., Ref. (16). However, a recent study describes a laboratory-based distinction between the two illnesses (17).

Recent ME/CFS reports brought optimism (18). National Institutes of Health in the US announced that it will prioritize the disease. Cornerstones are studies on PEM (12, 19) and effects of immunosuppressive treatment (20–22) although not substantiated in a phase III trial.

There are several partly competing explanatory models for ME/CFS, for example; autoimmunity, chronic infection, energy metabolic defect, imbalance in autonomous nervous system and/or hormones, and psychosomatic dysfunction. In this laboratory-oriented review, we present an overview of recent findings and attempt to bring a substantial portion of ME/CFS symptoms and its disease history into one explanatory model. The model draws analogies from more established autoimmune diseases (even if much remains to be understood in these too) is based on clinical experience and on recent immunometabolic results. Clues for further research are given in Table 1 and as separate statements in the text.

**Table 1:** Some outstanding questions regarding ME/CFS, which are addressed in this conceptual review.

The hypothesis gives rise to several verifiable general questions
What is the nature of the genetic predisposition? Can the infection history of ME/CFS patients be traced? Does it differ from those of other diseases, e.g., autoimmune ones? Is there a common sequence of infection, postexertional malaise, and comorbidity occurrence during ME/CFS pathogenesis? Can defects in tolerance development be detected in ME/CFS patients? Can the path of B cell clones from germ line to various autoreactivities be traced in ME/CFS patients? Which autoantibodies can be detected in ME/CFS patients and its comorbidities? Can clues to ME/CFS biomarkers be derived from this explanatory model?

## Trying to Place it all Under One Umbrella: a Hypothesis for ME/CFS Pathogenesis

We propose a pathogenetic model reminiscent of current thinking on the pathogenesis of autoimmunity.

**Figure 1:**
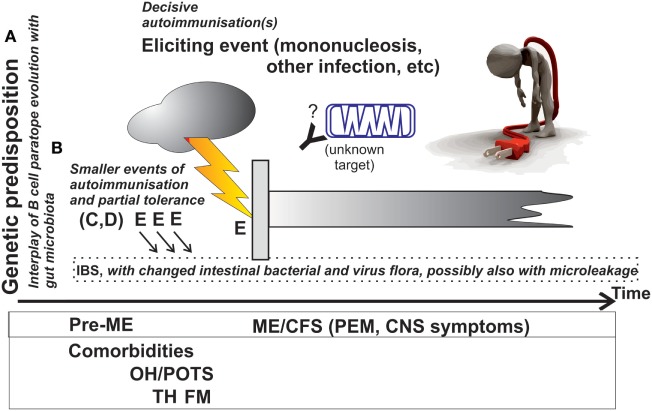
Approximate course of events during which ME/CFS develops, and overview of the explanatory model. The postulated immunometabolic energy block is shown as an antibody and a mitochondrion. Italicized text refers to the explanatory model presented under “Trying to place it all under one umbrella.” Abbreviations are explained in the text.

**Figure 2:**
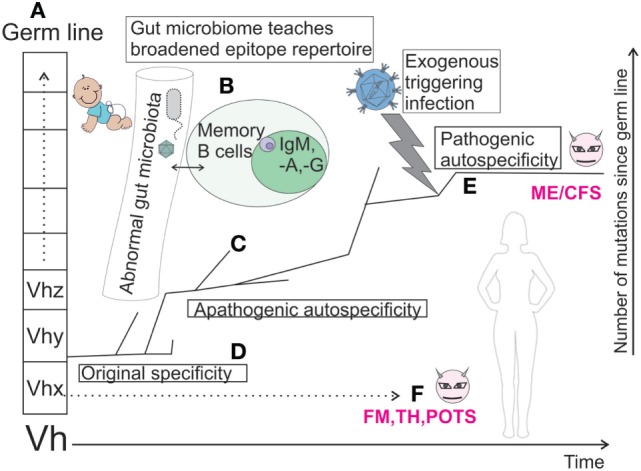
Mutational fate of a hypothetic germ line immunoglobulin heavy chain sequence (Vhy) in successive B cell clones, which gradually expand their paratope diversity in interplay with gut microbiota, T cells, and dendritic cells. If there is a chronic antigen stimulation, sequences more or less close to germ line sequence may be selected. Resulting B cells are stored as memory cells in germinal centers of gut-associated lymph nodes. Some of the developmental branches end due to clonal anergy or deletion (tolerization). Others are postulated to descend along a path to autospecificity due to an abnormality in gut commensal spectrum. An exogenous, triggering, antigenic stimulation (e.g., infection), eventually leads to overt pathogenic autospecificity (“evil” B cell clones, magenta) and ME/CFS. Similar fates of other B cell clones, which eventually turn autopathic and give comorbidities, are indicated under “F.” Characters A–F in bold refer to the stages mentioned under “Trying to place it under one umbrella.” This figure was inspired by work on the autoreactive clone VH4-34 (23, 24).

A genetically predisposed person (A) is exposed to successive infections (B), e.g., in the gastrointestinal tract—manifested as dysbiosis or irritable bowel syndrome (IBS)—or in the airways, with microbes carrying epitopes mimicking human self-epitopes, or microbes which activate autoreactive B cells to produce the so-called natural antibodies with non-rearranged germ line immunoglobulin genes. Such autoreactive B cells may be deleted or persist in a state of anergy (C). A proportion of these B cells remain in spleen and lymph nodes as memory IgM^+^, IgA^+^, or IgG^+^ B cells (D). Individuals differ in time and extent of encounters with autoreactivity eliciting microbes. Some encounters are here postulated to give rise to autoantibodies (E) against key enzymes in energy metabolism hence causing a defective aerobic energy metabolism and PEM, the central symptom of ME/CFS, others to fibromyalgia (FM), yet others to postural orthostatic tachycardia syndrome (POTS) or other comorbidities. If the autoimmunization events are independent of each other they can occur in any order. If there are cooperativity effects they may follow a rather specific order (F). The upper case letters refer to stages in Figures 1 and 2.

Thus, the basic property of ME/CFS patients would be a defect in tolerance coupled with a chance exposure to microbes carrying relevant mimicking autoantigen epitopes.

The italicized text of Figure 1 shows a hypothetical explanation of the events behind ME/CFS. A known function of microbes in the gut is to train, from within, the immune system to recognize and react correctly to microbes (including bacteria and viruses), which come from the outside (25–29). The correct reaction includes, among other things, anergy and unresponsiveness to microbial antigens that cross-react with self-antigens. It is known that ME/CFS patients often have IBS (30–32). In this IBS there is also a modified gut flora (33, 34). A less symptomatic gut dysbiosis may also occur (33, 35). In addition, there is also occasional epithelial barrier leakage of gut microbes. It is reasonable to assume that the innate mucosal immunity defenses have been breached or that peripheral tolerance maintenance (training function) of the gut flora has been disturbed. Normally, the mucosal immune system must maintain tolerance to harmless foreign antigens including food and commensal microbes. Presentation of antigens at mucosae often leads to tolerance (36). When there is microleakage tolerance may not function properly leading to loss of checkpoints that normally prevent development of autoreactivity (37–42). The profile of B cell subpopulations is different in ME/CFS compared with controls (43). A factor behind that could be new memory B cells with autoreactivity, which normally would be sorted out, arising and persisting. When the body is exposed to a new infection, these B cells could produce antibodies which react both to microbe and autoantigen. Autoantibodies and T cells that recognize self-peptides can damage cells which carry autoantigens. This is the so-called mimicry (antigen similarity) theory behind autoimmune disease (44). Part of the explanation for ME/CFS would then be the disturbed gut flora and microleakage from the gut. At the left side of Figure 1 is written “Genetic predisposition.” This is compatible with the increased frequency of ME/CFS in certain families. Like for many other common diseases ME/CFS could depend on both inheritance and environment.

If this hypothesis is correct, a tendency for autoreactivity would arise gradually, *via* a changed gut flora and microleakage from the gut. After a decisive immunization event autoimmunity leading to ME/CFS would arise, as shown in Figure 1. The prerequisites for autoimmunity would arise gradually because B cells with a tendency for autoimmunity would arise after recurring microleakage across the mucosal barrier of the gastrointestinal tract inducing a state of chronic inflammation. The normal contact between gut microbes and immune system occurs at the gut/mucosa interphase. Central tolerance often develops by elimination of autoreactive B cells. However, a proportion of autoreactive B cells remain which are kept unresponsive (anergic). When there is microleakage, the mucosal barrier is bypassed and tolerance may not be maintained. Autoreactive B cells can then be activated and differentiate to autopathic B cells.

The frequency of IBS, alterations in microbiome and extent of microleakage should be further studied in ME/CFS. Attempts to find autoreactive B cells to find their origin and their evolution should be made. Maybe it is possible to trace how they evolved by systematic sequencing of their antigen-binding structures (paratopes and idiotypes), from germ line to anti-gut microbe to autoimmune clone?

## Genetic Predisposition and Premorbid Phenotype

There is evidence for a strong genetic component in some autoimmune diseases, such as complement component deficiencies in SLE which may lead to reduced self-antigen elimination. Likewise, in ME/CFS, autoimmune diseases, for example, thyroid disease (45), SS (46), and SLE (47), often occur among relatives and sometimes among the patients themselves.

Presence of an HLA association is a hallmark of many autoimmune diseases. It indicates an aberrant immune presentation to either cytotoxic T cells (HLA Class I) or T helper cells (HLA Class II) which predisposes for autoimmunity. One study found an overrepresentation of HLA Class II DQA1*01, with an odds ratio of 1.93 (48).

Specific cytokine gene polymorphisms were observed; an increase of one, for TNFα, and a decrease of one, for IFNγ, were found in CFS (49).

Recent genome-wide association studies showed an increased frequency in ME/CFS of single-nucleotide polymorphisms (SNPs), some isolated, some concentrated to three gene regions: microtubule associated protein 7, CCDC7 (coiled-coil domain containing 7) and a T-cell receptor alpha chain gene (50). The latter may confine an increased tendency to autoimmunity. The comorbidity with autoimmune disease or disease having an increased prevalence of autoantibodies, e.g., FM (51–57), IBS (58–63), POTS (64), and hypothyroidism (45, 51, 55, 65–67), also indicate a tendency for autoimmunity in ME/CFS patients (further detailed in the section on autoreactivie B cell clones and autoantibodies, including Table 4). SS (46) and SLE (47) often occur among relatives and sometimes among the patients themselves.

IgG3 and mannose binding lectin deficiency were more common among ME/CFS patients than in controls (102, 103). IgG subclass deficiency is more frequent in ME/CFS than in controls (104, 105). Such deficiencies could increase the risk of recurrent infections.

In a genetic study concentrating on hormone and hormone receptor genes, certain TRPM3 and CHRNA2 SNPs were found to be more common in ME/CFS (106–108).

### Are There Also Epigenetic Changes in ME/CFS?

DNA modification (methylation) of promoters of some genes associated with immune cell regulation; glucocorticoid receptors, ATPase and IL6 receptor, respectively, was reported to differ between ME/CFS and controls (109). DNA methylation depends on the one-carbon metabolism, where ME/CFS changes have been recorded. Although the reason for such hypomethylation can only be speculated upon, it is interesting that the combined action of the vitamins B12 and folic acid play a fundamental role in providing methyl groups to hundreds of substrates in various elementary cell processes (see the section “can autoimmunity explain energy metabolic disturbances and PEM”).

### Gene Expression in ME/CFS

In a recent RNA-seq study, there were no specific RNAs expressed in ME/CFS compared with healthy controls and other chronic diseases (110). In another expression study, prominent differentially expressed genes were EIF4G1, EIF2B4, MRPL23, which control RNA translation, in cytoplasm and/or mitochondria (111). A differential expression of genes crucial for T-cell activation and innate response to viruses was also described (111–114) in CFS.

A novel angle was the report that the pattern in cerebrospinal fluid (CSF) and blood of another kind of RNA, the small regulatory RNAs, differed between ME/CFS, GWI, and controls (17, 115). Another pattern was found in FM (116). The pathophysiological roles of the small regulatory RNAs are still uncertain, but the findings indicate additional levels of pathophysiological regulation, which also could provide diagnostically useful biomarkers.

A prerequisite for calling a disease chronic is duration of at least 6 months. This often means that one has not been able to take samples during the period when the disease commenced. A common situation is that the patients remember that ME/CFS started with an infection, often infectious mononucleosis (IM), or a general virosis-like disease (117). When the acute infection with fever, myalgia, and swollen lymph nodes and/or cough subsides, a malaise and fatigability remains. According to the literature approximately 70% of ME/CFS cases start rather abruptly in this way. Others have a more gradual debut. The natural history of the disease should be studied systematically.

In a few cases, ME/CFS appear epidemically, with several cases being derived from a common index case. Even if epidemic outbreaks are uncommon it indicates that the disease might be contagious. Further epidemiological studies are needed.

## Many Different Infections have been Observed at the Outset of ME/CFS Like Disease

There is abundant evidence for infection as a trigger of chronic fatigue in a more general sense (often manifested as fatigability) (68, 72–74, 77–79, 82, 84, 118–128) (Table 2). But negative evidence also exists (129, 130). Some of this evidence is inconclusive (131, 132). Whether all these instances of postinfectious fatigability have identical properties (e.g., Do they fulfill criteria for PEM?; For ME/CFS?; How chronic are they?; etc.) should be systematically investigated. These infections can be traced in the patient history, by direct detection of the microbe(s) (133), or by detection of antibodies to the microbe(s) (94, 119, 133–146), see, however, Ref. (147).

**Table 2:** Long-standing fatigue, or fatigability, after an infection.

Microbe	Infection	Diagnostic term	Approximate % of fatigued post infection	Reference
Epstein–Barr virus	Infectious mononucleosis	Postviral fatigue	11% (6 months); 4% (12 months)	(68, 69)
*Coxiella burnetii*	Q fever	Post Q fever fatigue	10–20% (6–12 months)	(69–71)
*Giardia lamblia*	Giardiasis	Post Giardia fatigue	<1% (12 months)	(72, 73)
Ross River virus	Ross River virus infection	Post Ross River fatigue	11% (6 months); 9% (12 months)	(69, 74)
Chikungunya virus	Chikungunya virus infection	Post Chikungunya fatigue (often together with arthralgia)	20% over background (≥12 months)	(75, 76)
West Nile virus	West Nile virus infection	Post West Nile fatigue	31% (6 months)	(77–79)
Dengue virus	Dengue fever	Post Dengue fatigue	8% (2 months)	(80, 81)
Ebola virus	Ebola hemorrhagic fever	Post Ebola fatigue	Not clear, at least 10% (6 months)	(82, 83)
SARS corona virus	Severe acute respiratory syndrome	Post SARS syndrome	Approximately 22/400 = 6% (≥12 months)	(84)

How often does it happen that spouses are afflicted? This would advocate a transmissible factor rather than inheritance.

Epstein–Barr virus (EBV) seems to be a frequent trigger of ME/CFS (also referred to as “postviral fatigue”). Glandular fever (127), also called IM, is most frequently caused by EBV. A reasonably specific laboratory test for IM (the “Mono” test) is based on heterophilic antibodies, which bind to carbohydrate antigens on non-human erythrocytes (148–150). If infectious triggers of ME/CFS are investigated, a positive Mono test provides an often recorded marker. Other infections are often not diagnosed as objectively. EBV belongs to the herpes virus family. It can infect and remain latent in B cells. At primary infection, EBV triggers massive activation of multiple B cell clones each secreting monoclonal antibodies which are coded by immunoglobulin heavy and light (IGH and IGL) chain genes with unique variable [immunoglobulin heavy chain variable (IGHV) and IGLV] genes, with the so-called complementarity-determining regions. The result is a polyclonal B cell stimulation, with massive release of natural antibodies, including autoreactive antibodies. Besides B cell growth stimulation, EBV stimulates production of EBI3, one of two chains of the tolerance-control heterodimer cytokines IL27 and IL35 (151). Thus, EBV is deeply influencing immune functions. EBV is also coding for antigens with highly repetitive structure (e.g., Gly–Ala–Gly–Ala repeats in EBNA1). This may be a source for antigenic mimicry and development of autoreactivity. Both the B cell growth stimulation and such antigenic mimicry make EBV a prime suspect of inducing autoreactivity. There is a correlation between occurrence of IM and the autoimmune diseases MS (152–154) and SLE (155–158). How EBV is involved, be it frequent reactivations of latent EBV or defects in the T cell and NK cell surveillance mechanisms against the virus, is not clear, but the presence of EBV, and the immune response to it, should be compared in ME/CFS, MS, and SLE, see Ref. (134).

Summarizing, EBV is especially interesting as a facilitator of autoreactivity. Some autoantibodies may have an origin in a mimicry between EBV antigen and self-antigens. EBV is a ubiquitous virus. EBV can stimulate thousands of B cells to produce thousands of different antibodies, each with its own unique antigen-binding site. It often occurs as an eliciting factor triggering ME/CFS, in this case referred to as “postviral fatigue.” As mentioned, it stimulates growth of a wide variety of B cells, and it has viral proteins that can give rise to autoantibodies (159, 160).

Transmissibility is a microbial property. Most ME/CFS cases are sporadic (118). However, there are a few recorded outbreaks, where healthy ME/CFS patient contacts developed symptoms of the disease (118, 161), forming ME/CFS outbreaks in ME/CFS, indicating a transmissible agent.

Many observations support that a condition similar to ME/CFS occurs in approximately 10% of those who had Q fever, an infection with the bacterium *Coxiella burnetii* (69, 70, 74) which often occurs in outbreaks. Q fever is unevenly spread throughout the world. ME/CFS is more widespread. Q fever is therefore unlikely to be a common cause of ME/CFS, globally.

A chronic postinfectious fatigue/fatigability reminiscent of PEM occurs after a number of life-threatening virus infections (Table 2). There is not much antigenic similarity between these infective agents. Many infections which fundamentally challenge or reorganize the immune system give rise to a persistent, perhaps autoimmune, malfunctioning state.

Note that the number of ME/CFS cases triggered by severe zoonotic infections such as Ebola must be very small, on a global scale. Besides IM, mild respiratory infections like those caused by mycoplasma (162–168) and chlamydia (164, 169, 170), or general infections due to parvovirus B19 (136, 171) and herpes 6 and 7 (133), have been mentioned, although the diagnostic evidence is not strong.

Although much remains unclear before the role of infection in autoimmunity is understood, there are diseases where a known antigenic challenge triggers autoimmune disease, an infection (172) or a vaccination. Surprisingly often it is the brain that is the target for this autoimmunity (Table 3).

**Table 3:** Autoimmune syndromes secondary to infections–association/hypothetical relationship.

Disease	Microbe	Type of microbe	Mimicry (likely structure)	Reference
Postinfectious encephalitis	Measles, Varicella-zoster, etc.	Virus	Anti-myelin oligodendrocyte glycoprotein and unknown antigens	(85, 86)
Guillain–Barré syndrome	*Campylobacter* (primarily) and Zika virus	Bacterium and virus	Gangliosides; unknown antigen	(87, 88)
“Nodding disease”	*Onchocerca volvulus*	Worm	Unknown antigen	(89)
Pediatric autoimmune neuropsychiatric disorders associated with streptococcal infection (PANDAS)	*Streptococcus* infections, i.e., strep throat or scarlet fever	Bacterium	Carbohydrate antigens?	(90)
Multiple sclerosis	Epstein–Barr virus and other pathogens	Virus and bacteria	Myelin basic protein, proteolipid protein, and myelin oligodendrocyte glycoprotein	(91, 92)

In addition to commonly known microbes (virus, bacteria, and protozoa), a large number of new ones have been discovered during the last 5 years (171, 173–179). Many of them are viruses which do not cause any known disease. We need to keep an eye on these microbes. Maybe there are some among them which can precipitate ME/CFS?

In these examples of infection elicited autoimmunity, the microbial antigen mimics epitope(s) on human cells. Such microbial epitopes may either be small molecules, like lipids, or added to proteins posttranslationally (172), randomly similar sequences, repetitive sequence motifs, or highly conserved antigenic structures.

An example of the former mechanism (posttranslational antigenic modification) is PBC. The antibodies are directed against a small fatty acid molecule, lipoic acid, added posttranslationally to a protein in the pyruvate dehydrogenase (PDH) enzyme complex. PDH is part of the energy producing machinery at the surface of mitochondria (180, 181), and governs the transition from glycolysis (anaerobic energy metabolism) to the tricarboxylic acid cycle and respiratory chain (aerobic energy metabolism), occurring inside mitochondria. Lipoylation is a posttranslational modification, which also occurs in a few bacteria, such as *Novosphingobium* (182). Gut infection with *Novosphingobium* is a possible cause of PBC. The PBC patients have a PEM reminiscent of the PEM of ME/CFS. Likewise, periodontal infection with *Aggregatibacter*, which citrullinates its own as well as human proteins, may provide the final trigger for rheumatoid arthritis (183).

An example of the latter mechanism (conserved epitopes) is a family of highly conserved proteins, which are present in both humans and microbes, called “heat shock proteins” (HSP). Antibodies against HSPs occur in many often studied autoimmune diseases, for example, MS and SLE (94, 184–188). We found a higher frequency and levels of antibodies against a specific portion of HSP60 in ME/CFS patients (94). Even though HSP60 is a mitochondrial protein it is unknown if these antibodies can influence mitochondrial function.

## Immunological Aspects of ME/CFS

How does autoreactivity develop? Much remains to be learned. The adaptive portion of the immune system (B and T cells) has a formidable task, to distinguish “self” from “non-self,” i.e., autoantigens from antigens of invading microbes. After an infection the immune response is initially relying on players of the innate immune system with natural antibodies, receptors for pathogen-associated molecular patterns, danger-associated molecular patterns, and DNA sensors for exogenous pathogens. However, within a few weeks the immune system acquires a higher precision with the developing adaptive immune B and T cells ensuring that only the targeted microbe is destroyed. The target selection (and tolerance development) may go wrong. Some microbial targets are very similar to self-molecules. This is the basis for the so-called molecular mimicry theory, although the evolutionary lines for microbes and humans diverged long ago. There are still some structures, such as HSP, which have hardly changed at all since then. An immune defense against them thus constitutes a risk of promoting an autoreactive response.

So-called “natural antibodies,” which occur in all persons and mostly are of IgM nature, often both poly- and autoreactive, are produced by a CD20+, CD27+, and CD43+ subset of B cells (189). One function of these natural antibodies is scavenging of dead/apoptotic, damaged and infected cells. Only sometimes do they result in disease. In a healthy person, B cells that can produce autoantibodies often rest in an “anergic” state and do not produce their potentially damaging antibodies. They can be activated by the so-called “cell danger” signals. B cells which produce natural IgM have regulatory functions (190). Such “innate” immune cells which are on the border of auto- and alloreactivity may be starting points for development of autoimmune disease.

Disturbance in the composition of the gut microbiome, dysbiosis, has been detected in several diseases (33–35, 86, 191–195). A major function of the microbiome probably is to train the immune system (e.g., T cells, B cells, and dendritic cells) with a large variety of antigens. Disturbance in it may lead to a defective immune repertoire and imbalance of tolerance induction (196). As the tools for studying microbiota gradually become more precise, the possibility of more or less specific changes in microbiota predisposing to autoreactivity is increasingly being addressed. This is the case for type 1 diabetes (27, 197, 198), multiple sclerosis (199), rheumatoid arthritis (200), SLE (201), Behcet’s syndrome (202), autoimmune gastritis (203), and ankylosing spondylitis (204). ME/CFS patients also seem to have aberrations in their gut microbiota (33, 192, 205, 206).

A symptomatic variant of gut dysbiosis, IBS (207), a common comorbidity in ME/CFS, may influence mucosal tolerance induction. Indeed, ME/CFS with IBS was suggested to be a distinct subset of ME/CFS (208).

It is conceivable that if the mucosal barrier also is broken by microleakage (28, 34, 37, 41, 192, 209–210), tolerance development may become impaired, facilitating development of autoreactivity (37–42, 211–213). Autoimmunity often seems to be a hit and run phenomenon. However, a chronic underlying infection cannot be excluded, also in the ME/CFS case (214, 215).

The hypothesis presented in Figure 2 is based on findings regarding the IGHV gene sequence VH4-34 (23, 24) in SLE. It elaborates the immunoevolutionary aspect of Figure 1. Its explanatory model is similar to current thinking regarding the pathogenesis of autoimmune disease. It tries to clarify the genesis of autoreactive B cell clones from germ line to pathogenicity. The original specificity conferred to a B cell and immunoglobulin by VH4-34 is anti-branched lactosamine containing carbohydrates. This then gradually mutates, probably due to exposure to epitopes from commensal gut bacteria. The original specificity exists in the beginning of a mutational walk in the Vh genetic maze, an example of epistasis, where one mutation facilitates other mutations during avidity maturation. Other unknown antigenic stimulations then give rise to various autoantibodies, some with anti-DNA specificity. We hypothesize that pathogenic autoantibodies in ME/CFS are created by a similar mechanism. Thus, we postulate that a genetically predisposed person gets a deranged gut microbiome which gives rise to apathogenic B cells with a weak autospecificity. They are not eliminated due to a defective tolerance induction. Finally, these clones are given an antigenic stimulation from an exogenous infection which yields pathogenic B cell clones. Thus, memory B cell clones with a paratope spectrum derived from germline and subsequent exposure to commensal microbes, e.g., in gut, may be an important intermediary step before development of outright autoimmunity. It should be possible to follow the path to autopathic clones by isolation and sequencing the variable immunoglobulin chains in B lymphocytes in ME/CFS, like what was done with VH4-34.

### Autoreactive B Cell Clones and Autoantibodies in ME/CFS

Several autoantibodies have been found in ME/CFS (54, 93, 94, 216–222), and some of its comorbidities (Table 4). This is circumstantial evidence for ME/CFS being an autoimmune condition. Especially interesting are the results where an increased frequency of antibodies to certain hormone receptors was found (93). Several ME/CFS symptoms may be explainable by receptor interference from such autoantibodies.

**Table 4:** Occurrence of autoantibodies in ME/CFS and some of its comorbidities.^a^

Disease (frequency in ME/CFS), reference	Antigen to which autoantibody occurs more often than in controls					
	Phospholipid	Carbohydrate	Hormone	Hormone receptor	Ion channel protein	Other protein
ME/CFS	Cardiolipin (54)	Ganglioside (54)		β-Adrenergic and muscarinic cholinergic (93)		HSP60 (94)
Fibromyalgia (35–73%) (95)					Potassium channel transporter (96, 97)	
(hypo)Thyroidism (thyroiditis by cytology, 40%, wide definition of chronic fatigue) (98)			Thyroperoxidase (45)	Thyroid-stimulating hormone (99)		
Postural orthostatic tachycardia syndrome and/or orthostatic hypotension (27%) (100)				Acetylcholine (101)	Calcium channel transporter (101)	
Irritable bowel syndrome (35–90%) (30–32)						Vinculin and cytolethal distending toxin B (58)

*^a^The list is not complete. More studies are needed to obtain better statistics. Some of these autoantibodies have the potential to become diagnostic biomarkers. Abbreviations are explained in the text*.

In autoimmune conditions with pathological autoantibodies, erroneously activated and mutated B cells are the root of the evil (Figure 2). These should be studied in detail (43). One can envisage large scale sequencing of immunoglobulin gene variable domains of such clones to define aberrant specificities, with autoreactivity. A characteristic variation in B cell subsets (43) has been described in ME/CFS.

This should be studied systematically. At which time point did these diseases manifest themselves, before or after the ME/CFS started? How large is the frequency of autoantibodies in patients with these conditions, preferably measured simultaneously in an antigen matrix? Maybe there are autoimmunity biomarkers which could be used for ME/CFS diagnosis?

### Cytokine Patterns in Blood and CSF in ME/CFS

The immune system is engaged in ME/CFS (220). Several studies have found changes in cytokine pattern in blood and CSF, and in expression of cytokine genes (223–231), especially after exercise (232–238), concomitant with an increase in reactive oxygen species (ROS) levels and a decrease of HSP70 concentration (239), often in connection with a “flare,” an acute exacerbation of ME/CFS symptoms (237, 238). A difficulty is that cytokine patterns (Table 5) are inherently variable. The cytokine profiles may be different in different stages of the disease (225, 240).

**Table 5:** A selective list of cytokines whose concentrations were reported to change in ME/CFS.

Cytokine	Body fluid	Up- or downregulation	Reference	Comment
TGFα	Serum	+	(225)	
TGFβ	Serum	+	(226, 227)	Most consistent finding, although one inconclusive (241)
TNFα	Serum	+	(225)	Elevated early after debut
IFN-γ	Serum	+	(225)	Elevated early after debut
IL1α	Serum	+	(225)	Elevated in early stage of ME/CFS
Eotaxin-1 (CCL11)	Serum	−, +	(225, 226)	Positively correlated with severity and low early after debut
Eotaxin-2 (CCL24)	Serum	+	(223)	
Leptin	Serum	−	(230)	Inversely correlated with severity
IL13	Serum	+	(226)	Positively correlated with severity
IL6	Serum	+	(242)	Elevated early after debut
IL7	Serum	−	(223)	
IL8	Serum	+	(242)	Elevated early after debut
IL10	Cerebrospinal fluid	−	(228)	
IL16	Serum	−	(223)	
IL17A	Serum	+	(225)	Elevated early after debut
VEGFα	Serum	−	(223)	

A more permanent dysregulation of cytokines in plasma has also been reported (223, 225, 226, 228, 230, 243), see Table 5. A correlation with disease duration was seen (225, 242). A meta-analysis showed that an increased level of TGFβ in plasma in ME/CFS versus controls was the most consistent finding (227). Cytokines in CSF were also deranged in ME/CFS (224, 228).

Table 5 is a compilation from recent publications on cytokine abnormalities in ME/CFS. A recent meta-analysis concluded that many of the reported findings are not reproducible (227). This could reflect different levels of physical activity, the volatile nature of cytokine levels and methodological problems, such as collection, handling, and preparation of samples. There could also be a heterogeneity within the ME/CFS group which blurs the patterns, see, e.g., Ref. (224, 234).

Whether there are cytokine changes after exercise peculiar to ME/CFS is a related subject (244). A recent meta-analysis concluded that complement factor C4a split products, oxidative stress markers and leukocyte expression of IL10 and toll-like receptor 4 genes are reproducibly different from controls in ME/CFS (233). However, there may be subgroups within the ME/CFS group with radically different reactions to exercise. A clear-cut difference in gene expression after exercise between ME/CFS patients which have POTS comorbidity, and those who do not, was found (234).

The activity of the so-called natural killer cells is also changed (231, 244–248) in ME/CFS. However, a negative report came recently (249). The latter may be due to methodological differences. Both kinds of immune change (cytokines and immune cell activity) are potential biomarkers and should be studied more.

### Is There a General Defect in Tolerance Development in ME/CFS?

Tolerance induction is a major property of the gut mucosal immune system (196). A special kind of T helper cells, T_reg_, mediate mucosal tolerance, and anergy of tolerized B cell clones, *via* IL10 and TGFβ. It may be more than a coincidence that a change in TGFβ levels in serum was the most consistent cytokine change in ME/CFS versus controls (Table 5).

A defective tolerance development could in principle be detectable as a tendency to develop autoimmune disease in ME/CFS. The comorbidity between ME/CFS and better studied autoimmune disorders such as SS (250), SLE, and multiple sclerosis (251) is an indication of this. Fatigability, which may or may not be related to the PEM of ME/CFS (252–255), occurs as a major symptom in some autoimmune (6, 184, 256–261), mitochondrial (262, 263) and infectious (264) diseases. Immunostimulation, e.g., with Staphylococcal vaccine, theoretically could induce tolerance to autoepitopes involved in ME/CFS pathogenesis (265–267). It was reported to be effective in ME/CFS in a double-blind study (267). Symptom relief paralleled anti-staphylococcal antibody presence (266), arguing for impaired development of tolerance to autoepitopes of microbial origin in ME/CFS. Further studies are needed.

A strong argument for B cell-mediated autoimmunity in ME/CFS has been the rituximAab effect (20, 22). Around 60% of patients improved after a lag period. Rituximab is a monoclonal antibody directed against CD20, a surface antigen expressed on the majority of B cells. They are killed when the antibody binds to them. However, in a recent phase III trial there was no statistically significant effect observed (Mella, personal communication). Until results of the trial are published, it is not known whether this was due to a major placebo, or a minor rituximab, effect. CD20 is mainly present on B cells, but is neither expressed on immature B cells nor on most antibody producing cells such as plasmablasts and plasma cells (268). Part of the problem may be the subjective estimation of symptoms, prone to overestimation of placebo effects. In future double-blind studies of treatments for ME, objective symptom measures should be used to a larger extent. Another confounding factor may be heterogeneity within the ME/CFS patients although they were diagnosed according to the Canada criteria. Detailed studies are strongly recommended. Unpublished phase I and II studies have shown improvement in ME/CFS patients after treatment with the more unspecific immunosuppressant cyclophosphamide (Fluge, personal communication). It is another sign of autoimmunity contributing to ME/CFS.

In autoimmunity dependent on autoantibodies, the erroneously activated B cells are the root of the evil.

### Increased Frequency of Lymphomas in ME/CFS

Chronic immune stimulation increases the risk for B cell lymphomas. This happens in many autoimmune diseases. In accordance with the autoimmune hypothesis for ME/CFS presented here, CFS patients have a greater risk of B cell non-Hodgkin lymphomas, in particular marginal zone lymphoma (OR = 1.88, 95% CI = 1.38–2.57), compared with sex and age matched controls (269).

In summary, the evidence for autoimmunity in ME/CFS is indirect or circumstantial. It rests on the effect of immunosuppression (although unsubstantiated in a double-blind trial) of anti-CD20, comorbidities with known autoimmunity (thyroiditis, thyroidism) or possible autoimmunity (FM, POTS, IBS), probable improvement after immunostimulation, and an increased frequency of certain autoantibodies and of B cell lymphomas. Of the Witebsky–Rose criteria for autoimmunity (270), direct; transfer of disease by antibody, and indirect; transfer of disease by cells to SCID mice, induction of disease by autoantigen, identification of antibodies within lesions, genetic predisposition, autoantibodies or self-reactive T cells, a few (genetic predisposition and increased frequency of autoantibodies) are partially fulfilled. Much work remains.

## Can Autoimmunity Explain Energy Metabolic Disturbances and PEM?

The objective measurement of energy metabolism by repeated cardiopulmonary exercise testing revealed a defective aerobic energy production in ME/CFS (19). This is manifested as an abnormal fatigability. Fatigability, which may or may not be related to the PEM of ME/CFS (252–255), occurs as a major symptom in some autoimmune (6, 184, 256–261), mitochondrial (262, 263) and infectious (264) diseases. It remains to be studied how unique the PEM of ME/CFS is.

**Figure 3:**
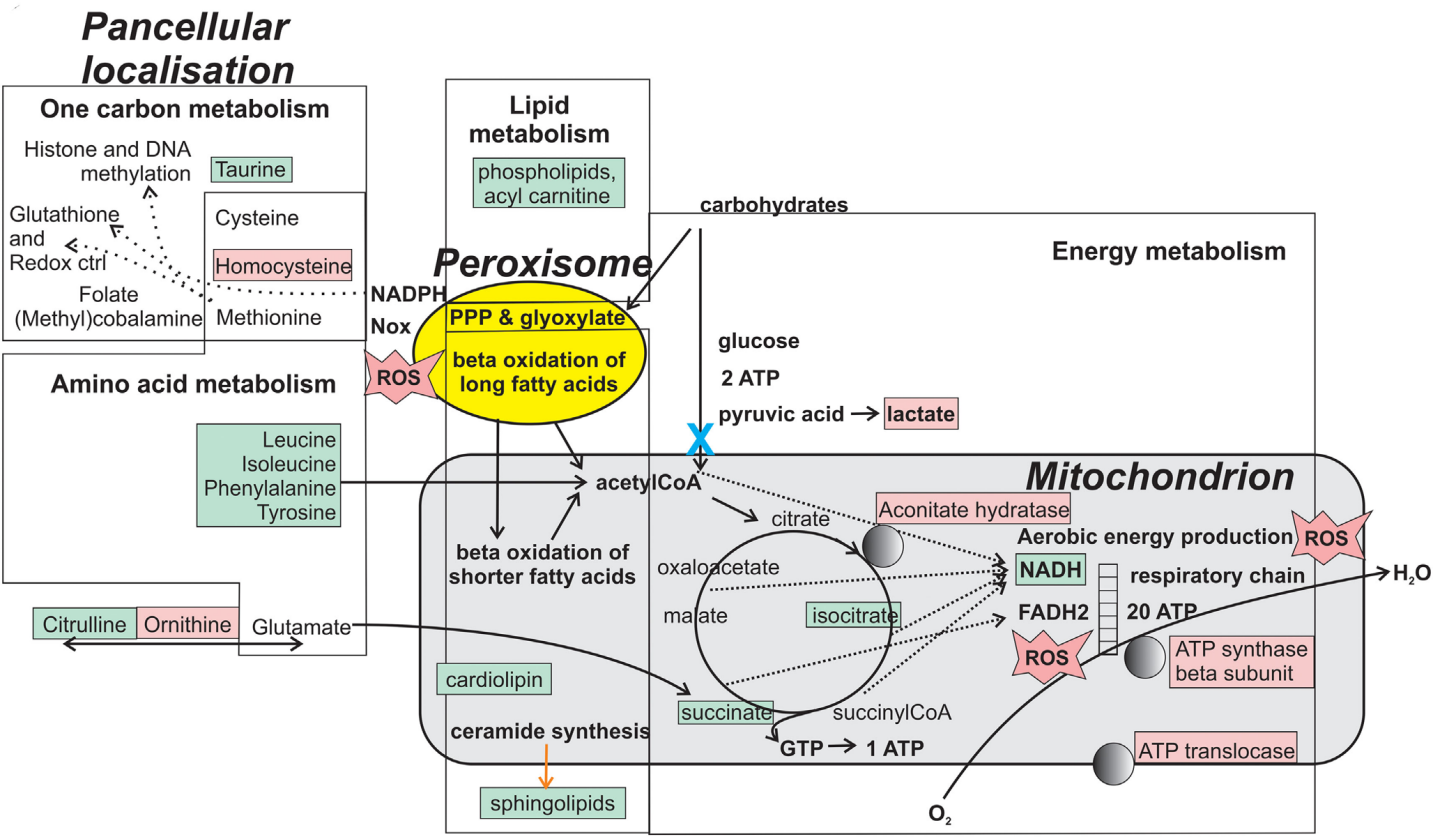
Metabolites and enzymes that are reportedly changed in ME/CFS. Molecules localized in energy metabolic organelles (peroxisome and mitochondrion), and the whole cell, are shown in pink if increased in abundance and green if decreased in abundance. Changes may sometimes be visible only after exercise. The blue “X” indicates a metabolic block implicated in ME/CFS (275). Normally functioning mitochondria convert oxygen to water through the respiratory chain. If the aerobic energy production is impaired, some oxygen can be converted to hydrogen peroxide and reactive oxygen species (ROS). PPP is the pentose phosphate pathway, an alternative pathway for energy production from carbohydrates. It produces the antioxidant NADPH. Together with glutathione, a product of one-carbon metabolism, NADPH controls ROS accumulation (“Redox ctrl”). A panel including some of the marked molecules may be useful as biomarker for ME/CFS.

Several observations indicate that the oxygen dependent (aerobic) energy metabolism is disturbed in ME/CFS (8, 19, 262, 271–273) (Figure 3; Table 6). That disturbance may be the reason for PEM. Mitochondria are the main producers of energy. They derive from α-proteobacteria which, over one billion years ago, were taken up into eukaryotic cells, see, e.g., Ref. (274). It is not unreasonable to guess that an immune defense against an infecting bacterium can cause collateral damage to mitochondria. While there must be protective mechanisms against this (e.g., tolerization), they may not always work.

**Table 6:** Potential energy metabolic biomarkers for ME/CFS.

Metabolic role	Metabolite or protein	Body fluid	Gain (+) or loss (−) in ME/CFS vs healthy controls	Reference	Comment
One-carbon metabolism	Taurine	Blood	−	(276)	
	Homocysteine	Cerebrospinal fluid (CSF)	+	(277)	

Oxidation	Reactive oxygen species (peroxide, etc.)	Serum	+	(239, 278)	Measured using thiobarbituric acid reactive substances

Amino acid metabolism (anaplerotic amino acids)	Leucine, isoleucine, phenylalanine, and tyrosine	Blood	−	(275)	

Urea cycle and amino acid metabolism	Citrulline	Blood and urine	−	(279)	
	Ornithine	Blood and urine	+	(279)	

Lipid metabolism	Phospholipids, including cardiolipin	Blood	−	(280)	
	Acyl carnitine	Blood	−	(276, 280)	
	(Glyco)sphingolipids	Blood	−	(280)	

Glycolysis	Lactate	Blood and CSF (muscle)	+	(271, 275, 281)	Higher after exercise (physical and mental)

Tricarboxylic acid cycle (TCA)	Isocitrate	Blood	−	(279)	

TCA	Succinate	Blood and urine	−	(282)	

TCA	Aconitate hydratase protein	Saliva	+	(283)	
	ATP synthase protein	Saliva	+	(283)	
	ATP translocase	Saliva	−	(283)	

A new dimension for understanding ME/CFS was added by recent publications (275, 279, 280, 282, 284). They revealed profound metabolic differences between ME/CFS patients and controls. Some of these changes may derive from an abnormal mitochondrial function in ME/CFS. Whether these abnormalities have an autoimmune origin is not known.

### Evidence for Inhibition of Key Energy Metabolic Processes in ME/CFS

A number of reports indicate a metabolic disturbance, indicative of mitochondrial dysfunction (19, 273, 275, 276, 279, 280, 282, 284–286) in ME/CFS. Evidence points to a defective aerobic energy metabolism. The aerobic energy metabolism (TCA + respiratory chain) gives an around 10-fold higher yield of ATP per glucose molecule than the anaerobic metabolism. There are similarities with PBC, a model of autoantibody mediated energy blockade (180, 287–292). In analogy with PBC, where IgG were found to be energy inhibitory, circulating energy inhibitors have been found in ME/CFS (275), although their molecular nature is unknown. The demonstration of such inhibitors has the potential to explain the disease and create efficient diagnostic tests. It would be logical if, like in PBC, these circulating inhibitors turned out to be immunoglobulins, presumably directed against mitochondrial proteins.

It is an important research task to compare PEM of PBC with the PEM of ME/CFS, and PEM-like fatigability in other diseases.

Fibromyalgia is a common comorbidity of ME/CFS, which also occurs in several established autoimmune conditions (55, 293–298). The delineation of ME/CFS from FM is sometimes not straightforward. FM muscle displays metabolic abnormalities (299, 300) reminiscent of those observed in ME/CFS. Besides the comorbidity, there seem to be both common (myalgia, muscle metabolic abnormalities, increased frequency of autoantibodies) and distinct (PEM, cognitive disturbance) aspects of these conditions.

### Can a Defective Energy Metabolism Also Explain the Cognitive Disturbances?

A deficient energy supply may also cause cognitive disturbance in ME/CFS (195). It can be elicited by both physical and mental (301, 302) activity. In analogy with accumulation of lactate in serum and muscle after exercise, increased concentrations of lactate in CSF have been found in ME/CFS (281, 303) and the related condition GWI (304). ME/CFS-specific changes in the CSF proteome which included accumulation of complement components, a sign of antibody activity, were also described (305).

Homocysteine is part of the one-carbon metabolism, which was reported to be deranged in ME/CFS patients, perhaps as a compensation for other energy metabolic disturbances. Homocysteine levels in CSF are a widely used marker of reduced cognition. In 1997, an investigation of homocysteine and vitamin B12 in CSF of patients who fulfilled the criteria of both FM and chronic fatigue syndrome was carried out. In comparison with a large healthy control group, all eleven patients in the study had increased homocysteine levels in CSF, although the blood levels were usually not increased. The CSF-B12 level appeared to be generally low. The high CSF-homocysteine and low CSF-B12 levels correlated significantly with ratings of mental fatigue. The results were at the time interpreted as suggesting a block of inflow over the blood brain barrier of B12 and/or folic acid (277). The derangement in one-carbon metabolism is supported by 20 years’ experience of vitamin B12 and B9 treatment in ME/CFS patients, which tends to diminish impaired cognition (“brain fog”) (306). It is not immediately evident why the one-carbon metabolic pathway would change after a block of aerobic energy production. The genesis of this metabolic aberration in ME/CFS should be further studied.

The state of the one-carbon metabolism also has profound epigenetic consequences. Both DNA and histone methylation depend on the availability of *S*-adenosyl-methionine.

### How Are Metabolic Disturbances Related to the Flare after Exercise?

The “flare” is a central event after exercise, accounting for much of the malaise in PEM.

A link between mitochondrial dysfunction and innate immune dysregulation is suggested by recent immunometabolic findings which demonstrate that the energy producing organelles (mitochondria and peroxisomes) are coupled *via* mitochondrial antiviral signaling protein, a signaling molecule, to the inflammasome, which can orchestrate release of inflammatory cytokines (307–314). Another sign of mitochondrial derangement in ME/CFS is the occurrence of ROS in serum, measured as increase in thiobarbituric acid reactive substances or decrease of reduced ascorbic acid (239, 278). These tests may also become part of a biomarker panel for ME/CFS. It was recently shown that oxidation of a critical cysteine residue in pyruvate kinase M2, one of the enzymes of the pyruvate dehydrogenase complex (PDC), can lead to a block in pyruvate production, potentially mimicking an autoimmune block of PDC activity (315, 316). Thus, although the pattern of metabolic changes in ME/CFS is compatible with a PDH block (275) (blue X in Figure 3), possibly of autoimmune origin, the block could also be caused by ROS (317), frequently increased in ME/CFS. ROS are produced in four places in the cell; NADPH oxidase (in Figure 3), peroxisomes and in respiratory chain complexes I and III (318). ROS production can be evoked by starvation (319) and respiratory complex I malfunction (320). ROS influence glutathione levels and indirectly the whole one-carbon metabolism. It could be a key player in ME/CFS pathogenesis. The origin and pathobiology of ROS in ME/CFS should be investigated.

## How Well do Clinical and Laboratory Data Fit into the Explanatory Model?

As shown in Table 7, much work remains before the autoimmune nature of ME/CFS can be considered established.

**Table 7:** How do recent findings fit into the explanatory model?

Proposed step	Finding	Degree of fit with presented explanatory model
Genetic predisposition	GWAS: ME/CFS-specific single-nucleotide polymorphisms in microtubule associated protein 7, CCDC7, and TCRα (50) HLA: increase in DQA1*01 (48) IgG subclass deficiency (102–105)	Imperfect, needs deeper study Presence of transmissible agent not excluded
Changes in microbiota	Reduced overall diversity (34, 191, 192) Divergence more concentrated to certain taxa (33, 35)	Imperfect, needs deeper study
Gut microleakage	Increased lipopolysaccharide (LPS), LPS-binding protein, and sCD14 in blood (34, 192)	Imperfect, needs deeper study
Autoantibodies	Yes, but maybe not disease specific (see Table 4)	Imperfect, needs deeper study
Triggering antigenic challenge	Epstein–Barr virus infection is a common trigger, some other infections also (see Table 2)	Imperfect. Retrospective diagnosis of infections is often problematic
Autopathic B cell clones	Removal of B cells by anti-CD20 or other immunosuppressants improves 50–60% of ME/CFS patients in phase I–II trials (20–22) Increased frequency of B cell lymphomas in ME/CFS patients (269)	Larger study with as objective measures as possible is necessary. Autologous bone marrow transplantation could give additional evidence
Defective tolerization of autoreactive B cell clones	Increased frequency of autoimmune disorders and comorbidities in ME/CFS patients Effect of microbial immune modulation (266, 321, 322)	Imperfect, needs deeper study
Disturbance of energy metabolism	Clear evidence of energy metabolic disturbance (275, 276, 279, 280)	Imperfect. Needs more observations, especially with reference to exercise
Autoimmunity causing energy metabolic disturbance	Circulating energy inhibitory factors demonstrated (like in primary biliary cirrhosis) (275, 292)	Molecular nature of inhibitors is unknown. If they are immunoglobulins, can they reach intracellular targets?

## Which Facts Do Not Fit into the Explanatory Model?

Even if ME/CFS is of autoimmune origin, is it the metabolic block (275) or the autoantibodies to hormone receptors (93) which are most important for pathogenesis?

The mechanism behind the flare after exercise (238) is obscure. Maybe a mitochondrial defect can lead to an increased activity in the innate immune network.

The disturbance in one-carbon metabolism (277, 280) may or may not be related to the disturbed transition between glycolysis and TCA cycle. It is indicative of a wider metabolic derangement than a block of PDH (275) would be expected to lead to. There are several papers on hormones (322), including glucocorticoids (257) and transient receptor potential channel hormones (222), and their receptors (106, 107, 109), in ME/CFS. It is conceivable that parts of the autonomic dysfunction can be explained in this way.

## Conclusion

ME/CFS is a challenge for the patients, for medical research and ethics, for all of public health, and for society. The intensified hunt for scientific evidence explaining ME/CFS has large consequences for many thousands of people. Many of the published results need repetition. But as shown in this article the signs that autoimmunity and energy metabolic deficiency is involved in the disease have increased. A hypothetical but logical path, from gastrointestinal tract dysbiosis, to formation of pathogenic autoimmune B cells, to inhibition of energy production and deficient cognition, to flares of cytokine production, can be delineated. The natural history indicates that in many cases infections can elicit or worsen this autoimmunity.

The recently intensified research on ME/CFS yielded many biomarker candidates, as mentioned in this study. The main consequence of this work is that the proposition that there is no logical somatic explanatory model for ME/CFS (14) can be refuted. However, like for virtually all autoimmune diseases, the explanatory model has several tentative steps which need further exploration. The elucidation of the molecular nature of circulating metabolic inhibitors in ME/CFS (275) is a central question. If they turn out to be immunoglobulins, they may directly yield diagnostically useful biomarkers and an explanation of the mechanism underlying ME/CFS.

The risk of giving a hypothetical unifying explanation, as in this study, is that hypothesis can be perceived as fact, and that it influences the perception of the disease. But contacts with ME/CFS patients and those who care for them have convinced us that most can handle the uncertainty that hypotheses involve. Without hypotheses we cannot direct the acquisition of further knowledge of ME/CFS.

## Author Contributions

JB conceived of the paper and wrote most of it. C-GG added substantial parts especially regarding the clinical aspects. AE participated in the writing. She is writing a book on ME/CFS, her comprehensive knowledge was valuable. MR participated in the writing. He concentrated on checking references. AR contributed substantially, especially regarding the immunological aspects.

## Conflict of Interest Statement

The authors declare that the research was conducted in the absence of any commercial or financial relationships that could be construed as a potential conflict of interest.
